# Genome Sequence of Klebsiella pneumoniae YBQ, a Clinical Strain Isolated from the Sputum of a Patient with Severe Pneumonia

**DOI:** 10.1128/MRA.01223-20

**Published:** 2020-11-19

**Authors:** Jintao Zou, Zhifu Chen, Xiaoli Zhang, Yue Yuan, Haiming Jing, Quanming Zou, Jinyong Zhang

**Affiliations:** a National Engineering Research Center of Immunological Products, Department of Microbiology and Biochemical Pharmacy, College of Pharmacy, Third Military Medical University, Chongqing, People’s Republic of China; b Department of Clinical Hematology, College of Pharmacy, Third Military Medical University, Chongqing, People’s Republic of China; Queens College

## Abstract

We report here the genome of Klebsiella pneumoniae YBQ, a clinical strain isolated from the sputum of a patient with acute Klebsiella pneumoniae infection. The genome consists of a 5,119,471-bp circular chromosome and a 184,347-bp plasmid. Genome annotation predicted 5,028 coding DNA sequences (CDSs), 84 tRNAs, 25 rRNAs, and 47 small RNAs (sRNAs).

## ANNOUNCEMENT

Klebsiella pneumoniae is an important opportunistic pathogen that frequently causes life-threatening infections, including pneumonia, bacteremia, and urinary tract infection (1). It accounts for up to 10% of all nosocomial bacterial infections (2) and is associated with high mortality (3, 4). Recently, due to the steadily increasing frequency of the subset of highly invasive and hypervirulent K. pneumoniae (hvKp) and carbapenem-resistant K. pneumoniae (CRKP) strains (5), infections caused by this bacterium have become a major threat to public health.

hvKp usually exhibits a hypermucoviscous phenotype, which is associated with high expression of extracellular polysaccharides (6). Recently, we isolated a clinical strain of *K. pneumoniae*, named YBQ, from the sputum of a pneumonia patient with acute and severe K. pneumoniae infection (ethics approval granted by the Ethical and Experimental Committee of the Army Medical University). A string test indicated that YBQ exhibits a hypermucoviscous phenotype (7). Animal challenge studies indicate that YBQ is a strain with high virulence (8). Here, we report the complete genome sequence of YBQ, which may be essential for further study of the virulence and pathogenic mechanism of this isolate.

A single colony of YBQ was picked and grown for 6 h in 20 ml LB medium at 37°C. Genomic DNA was isolated using the DNeasy blood and tissue kit (Qiagen), evaluated with gel electrophoresis, and quantified with a 2100 NanoDrop spectrophotometer. A SMRTbell template was used to establish a standard library of 20-kb fragments, and the genome was sequenced by single-molecule real-time (SMRT) sequencing using the PacBio RS II platform. PacBio subreads less than 1 kb long were removed. Draft genomic unitigs, which are uncontested groups of fragments, were assembled using Canu v1.5 (https://github.com/marbl/canu/releases/tag/v1.5). The total number of reads sequenced with the PacBio SMRT platform is 8,764,210, with 158,211 subreads and a subread *N*_50_ value of 12,359 bp. To improve the accuracy of the genome sequences, the GATK (https://www.broadinstitute.org/gatk/) and SOAP (SOAP2, SOAPsnp, SOAPindel) tool packages were used to make single-base corrections. To trace the presence of any plasmid, the filtered Illumina reads were mapped using SOAP to the bacterial plasmid database (http://www.ebi.ac.uk/genomes/plasmid.html).

Gene prediction was performed on the YBQ genome assembly by GLIMMER v3.02 with hidden Markov models. tRNA, rRNA, and small RNA (sRNA) recognition was performed using tRNAscan-SE v1.3.1 (9), RNAmmer v1.2 (10), and Rfam v9.1 (11). CRISPRfinder v4.2.19 was used for CRISPR identification (12). The final assemblies of YBQ resulted in a 5,119,471-bp circular chromosome and a 184,347-bp circular plasmid, with mean G+C contents of 57.67% and 50.47%, respectively. The total genes predicted for YBQ contain 5,028 CDSs, 84 tRNAs, 25 rRNAs, and 47 sRNAs, along with 2 CRISPR arrays. In addition, 118 tandem repeats were obtained using the Tandem Repeat Finder v4.04 (13); 58 minisatellite DNAs and 9 microsatellite DNAs were selected based on the number and length of repeat units.

Seven databases, including KEGG (14), Clusters of Orthologous Groups (COG) (15), nonredundant protein database (NR), Swiss-Prot (16), Gene Ontology (GO) (17), TrEMBL (18), and eggNOG (19), were used for general function annotation. The summary of the functional annotation of the genome is listed in Table 1.

**TABLE 1:** Statistical results of the functional annotation of the genome of YBQ

Database	No. (%) of genes annotated
KEGG	3,658 (72.75)
InterPro	4,567 (90.83)
SwissProt	3,617 (71.93)
COG	4,271 (84.94)
GO	3,530 (70.2)
NR	5,021 (99.86)
Total	5,023 (99.9)

### Data availability.

The annotated complete genome assembly of K. pneumoniae YBQ was deposited in the SRA. The BioProject accession number is PRJNA663465, and the SRA accession number is SRX9123846.
